# Intersection-Based Link-Adaptive Beaconless Forwarding in Urban Vehicular Ad-Hoc Networks

**DOI:** 10.3390/s19051242

**Published:** 2019-03-12

**Authors:** Khaleel Husain, Azlan Awang, Nidal Kamel, Sonia Aïssa

**Affiliations:** 1 Department of Electrical and Electronic Engineering, Universiti Teknologi PETRONAS, 32610 Seri Iskandar, Perak, Malaysia; khaleelhusain@ieee.org (K.H.); nidalkamel@utp.edu.my (N.K.); 2 Institut National de la Recherche Scientifique (INRS), University of Quebec, Montreal, QC H5A 1K6, Canada; aissa@emt.inrs.ca

**Keywords:** link duration, remote monitoring, receiver-based, road intersections, vehicular ad-hoc networks, winner relay management

## Abstract

Remote monitoring applications in urban vehicular ad-hoc networks (VANETs) enable authorities to monitor data related to various activities of a moving vehicle from a static infrastructure. However, urban environment constraints along with various characteristics of remote monitoring applications give rise to significant hurdles while developing routing solutions in urban VANETs. Since the urban environment comprises several road intersections, using their geographic information can greatly assist in achieving efficient and reliable routing. With an aim to leverage this information, this article presents a receiver-based data forwarding protocol, termed Intersection-based Link-adaptive Beaconless Forwarding for City scenarios (ILBFC). ILBFC uses the position information of road intersections to effectively limit the duration for which a relay vehicle can stay as a default forwarder. In addition, a winner relay management scheme is employed to consider the drastic speed decay in vehicles. Furthermore, ILBFC is simulated in realistic urban traffic conditions, and its performance is compared with other existing state-of-the-art routing protocols in terms of packet delivery ratio, average end-to-end delay and packet redundancy coefficient. In particular, the results highlight the superior performance of ILBFC, thereby offering an efficient and reliable routing solution for remote monitoring applications.

## 1. Introduction

Remote monitoring of vehicles moving in urban traffic environment significantly contribute to various applications in the field of telemedicine, road safety and mobile surveillance. Remote monitoring applications for road traffic environment generally involve data transfer from a moving vehicle to a static remote infrastructure. The data to be transferred include still images, signals from various physiological sensors, audio and video. Vehicular ad-hoc network (VANET) aims at achieving intelligent transportation system (ITS) and offers an ideal platform to enable remote monitoring applications in road traffic environment. In such network, all vehicles are equipped with wireless sensors and onboard units to allow wireless communication between the vehicles and the infrastructure [1]. Communications in VANET can be broadly classified into four types: in-vehicle, vehicle-to-vehicle (V2V), vehicle-to-infrastructure (V2I) and more recently vehicle-to-broadband cloud (V2B) communication [2]. Wireless access in vehicular environments (WAVE) [3] architecture comprising the IEEE 802.11p and IEEE 1609.x communication standards is used in VANETs. IEEE 802.11p is an approved amendment of IEEE 802.11 [4] focusing mainly on the physical and medium access control (MAC) sublayers whereas IEEE 1609.x comprises various communication standards that operate in the middle layers of the protocol stack to support safety applications.

Wireless communication required to facilitate remote monitoring applications in urban traffic environment faces significant challenges. Specifically, it has to cope with general vehicular environment constraints such as mobility and density variations [5,6,7]. In addition, urban traffic environment has its own constraints such as drastic speed decays, presence of road intersections and traffic signals [8,9]. Apart from this, wireless communication also needs to support different data rate requirements for various data types such as images, physiological signals, audio and video that need to be transferred in remote monitoring applications. The above mentioned constraints give rise to significant hurdles while developing routing solutions in urban VANETs. Routing protocols that use geographic information and make routing decisions on the fly are well suited for VANET environment. One of such routing techniques is the receiver-based data forwarding, where the intermediate vehicles upon data reception, decide whether or not to forward the data packet. The decision is usually based on certain characteristics of intermediate vehicle, previous relay vehicle and destination, with the most commonly used characteristic being geographic information. Receiver-based data forwarding techniques mainly comprise two key features: forwarding zone and waiting time criteria. Forwarding zone criterion limits the number of vehicles eligible for data forwarding based on their presence in the forwarding zone. Eligible vehicles then contend for the forwarding rights by setting their timers based on a certain waiting time criterion. A vehicle with the shortest waiting time wins the contention and forwards the data packet, while other contending vehicles cancel their timer and discard the data packet upon reception of duplicate data packet.

Although receiver-based schemes are well suited for dynamic environments, they result in significant end-to-end (ETE) delay [5]. This is due to their waiting time criterion, where the relay vehicles are required to wait for certain time for each data packet before forwarding it further. The work in [10,11] minimize this issue by allowing a relay vehicle that wins the contention to directly forward any incoming data packet without any waiting time for certain duration. This duration is usually based on the position and speed information of the vehicles. This helps in reducing the ETE delay, as relay vehicles do not have to perform relay selection for every incoming data packet. However, if the duration for which relay vehicle acts as a default forwarder is too long, the chances of a relay vehicle moving out of the forwarding zone will be high and may lead to multiple relay vehicles forwarding redundant data packets at the same hop level. In addition, the urban traffic environment comprises several road intersections leading to roads with different directions, and a relay vehicle acting as default forwarder for long time can lead to data packets being forwarded in the wrong direction, hence resulting in either packet drops or longer routes to destination. Therefore, assigning an appropriate duration for the relay vehicles to stay in a default forwarding state is vital and using the geographic information of road intersections present in urban VANETs can greatly contribute towards solving the issue.

This article proposes a receiver-based data forwarding protocol that uses geographic information of road intersections to assign a duration for which a relay vehicle stays in default forwarding state. The key contributions of the article are as follows:

- A novel receiver-based data forwarding protocol called intersection-based link-adaptive beaconless forwarding for city scenarios (ILBFC) is proposed. ILBFC exploits geographic information of road intersections in limiting the duration for which a relay vehicle stays as a default forwarder. Also, the maximum duration for which a relay vehicle can be a default forwarder is fixed based on the maximum road length between the two road intersections.
- An urban road traffic adaptive winner relay management scheme is employed, where the relay vehicle when acting as default forwarder decides on every data packet reception whether or not to continue as default forwarder. The decision is based on the relative geographic progress of current and previous relay vehicles towards destination. This is done with the aim to take into account the drastic decay in the speed of vehicles due to the road intersections and traffic signals encountered in urban VANETs.
- The proposed ILBFC is then simulated in realistic traffic conditions and its performance is compared with other existing state-of-the-art routing protocols. The comparison is done in terms of three performance metrics: packet delivery ratio (PDR), average ETE delay, and a new metric called packet redundancy coefficient (PRC), where PRC gives a measure of routing protocol’s ability to disseminate the data packet in unicast fashion.

The rest of the article is organized as follows. Section 2 gives the background information highlighting various remote monitoring applications, characteristics of remote monitoring in urban environment, different aspects of routing, and an overview of receiver-based data forwarding in VANETs. Section 3 explains all the features of the proposed ILBFC protocol in detail. Section 4 analyzes the performance of ILBFC in comparison to routing protocols from existing state-of-the-art. Specifically, this section details the scenario setup, the performance metrics, and the routing protocols used in the comparisons, and provides an in-depth discussion of the simulation results. Finally, Section 5 concludes the paper.

## 2. Background

In this section, background information is provided. Firstly, an overview of remote monitoring in urban VANETs is given followed by a review of some important characteristics and design challenges of the routing solutions for VANETs.

### 2.1. Remote Monitoring Applications in VANETs

Remote monitoring applications in VANETs can be broadly categorized into emergency telemedicine, commercial driver safety, and mobile surveillance. The scope of these applications in vehicular networks is briefly explained next.

#### 2.1.1. Emergency Telemedicine

Emergency telemedicine makes use of emerging wireless communication and information technologies to provide health-care expertise such as medical diagnosis, treatment and patient care, in remote sites [12]. Early medical diagnosis and treatment could be vital in patient’s recovery or even saving patient’s life. In addition, transmission of patient’s medical data from ambulance to the hospital allows the doctors to make necessary arrangements for the incoming patient. The medical data sent includes still images of patient such as X-ray report, medical video of the patient and various signals from physiological sensors such as electrocardiogram (ECG), electroencephalogram (EEG), heart rate (HR) and blood pressure.

Telemedicine projects in [13,14,15] aimed at developing an emergency telemedicine device to transmit important medical data such as still images and various signals from physiological sensors from a remote site to an emergency health-care center to enable prehospital assistance from doctors. Apart from this, a telestroke ambulance prototype was developed in [16] to enable realtime audio-video communication between the ambulance and the hospital site using cellular technology. The use of video technology enables doctors to perform more accurate diagnosis by visualizing different features of the patients such as colouring, rapid breathing, muscle stiffness, and state-of-shock evidence [17]. The work in [18] highlights a detailed explanation on various emergency telemedicine applications.

#### 2.1.2. Commercial Driver Safety

In [19], it was reported that commercial drivers often involve in more road accidents as compared to those who drive their personal vehicles. According to the studies by the social security organization (SOCSO), there were at least 2 work-related driving deaths in 2011. Also, there is a rise of almost 49% in work-related driving accidents from 17,682 accidents in 2007 to 26,262 in 2012 [20]. In [21], as part of Shell exploration and production hearts and minds research programme, four operating companies across Holland, Nigeria, Thailand and Sultanate of Oman took part in a study analyzing the commercial drivers’ attitudes in oil and gas industry that lead to risk-taking behaviour. Also, it was further reported that 50% of all fatalities in Shell’s exploration and production were related to driving.

Commercial drivers often involve in risk-taking behaviours as they are not monitored [21,22]. Additionally, since the drivers do not own the vehicle, they do not bear any financial burden in case of vehicle crash, which results in less responsible and more risky driving [23,24,25]. According to [26], half of the workers involved in vehicle crashes in the United States of America were either not using safety belts or were in ejected state from the vehicle. Fatigue could be defined as a risk factor between sleepiness and reduced vigilance among drivers, and it has been reported in [27] that commercial drivers are more involved in sleepiness as compared to drivers using their personal vehicles. This is because most of the commercial drivers tend to perform odd jobs at night while also driving at daytime and, hence, do not have enough sleep. In Malaysia, for instance, most of the fatigue-related fatal crashes were caused by heavy-vehicle drivers [28].

Video monitoring of commercial drivers while driving can contribute to improving road safety and can be used to caution the drivers and other vehicles on the road should there be any occurrence of unusual behaviour. According to the study carried out in [22], the use of driver monitoring resulted in an estimated average of 20% accident reduction. Moreover, drivers when aware of being monitored tend to change their attitude. Video monitoring can also assist in fatigue detection [29,30,31] by observing drivers’ various physiological features such as eye blinking, yawning and other movements.

#### 2.1.3. Mobile Surveillance

Remote monitoring in VANETs also has scope in mobile surveillance applications. Cameras can be mounted on top of buses, police vehicles and other government vehicles to collect the video surveillance data and transfer it to a monitoring center such as a police headquarter [32]. An embedded wireless video surveillance system for vehicles was created in [33] using 802.11-based WLANs and CDMA connectivity solutions. The video surveillance data was then accessible through the Internet. In [34], the performance of IEEE 802.11p vehicular video surveillance system was analyzed, and a mechanism for adaptive target video bit rate selection was proposed.

### 2.2. Specific Characteristics of Remote Monitoring in Urban VANETs

Wireless communication for the remote monitoring applications in an urban environment has unique characteristics. Some of the characteristics specific to remote monitoring in urban VANETs are explained next.

#### 2.2.1. Urban Environment Constraints

In general, the vehicular environment has unique characteristics that pose considerable challenge for efficient communication [5,6,7]. This environment consists of both stationary roadside units (RSUs) and mobile vehicles, and the mobility in this environment can vary. The mobility tends to be high in the case of fast moving vehicles and slow when the vehicles are moving at low speed. Both extreme cases of mobility have their own challenges. For high mobility scenarios, the wireless communication among vehicles has to circumvent issues such as frequent link failures, Doppler effect, large ETE delay, etc. For the low mobility scenarios, issues such as high packet collision, channel fading, interference, etc., become considerable. Apart from this, there exist significant density variations in vehicular environment, with high density being observed usually during peak hours or in case of traffic jams and low density when few vehicles are moving around. Density variation may pose various constraints for efficient communication among vehicles. High density scenarios face the issue of duplicate packet reception due to redundant messages sent by multiple vehicles. In contrast, low density scenarios face the issue of packet drop due to the unavailability of vehicles for further forwarding the message. Another characteristics of the vehicular environment is the constrained movement of vehicles due to the predefined paths (roads). Thus, location information of the paths can be used to further improve the efficiency of the wireless communication in this context. In addition to the general constraints of the vehicular environment, the urban environment has its own unique characteristics. The work in [8] demonstrated various parameters that have a significant impact on network performance in an urban environment. Vehicles in this environment face drastic speed decay due to the presence of road intersections and traffic signals. Therefore, the average velocity of a vehicle has minimum impact on the network performance as it does not reflect the actual velocity in urban traffic [8,9].

#### 2.2.2. Data Rate Requirement

As highlighted in Section 2.1, the type of data monitored for remote monitoring applications includes images, various signals from physiological sensors, audio, video, etc. To enable remote monitoring of these data through communication networks, different data rates are required. Table 1 highlights the data rate requirements of various sensors used by remote monitoring applications.

### 2.3. Routing in VANETs

The routing protocol in VANETs uses intermediate vehicles as relays to transmit the data packets from a source vehicle to the destination.

#### 2.3.1. Classification

In [5], the routing protocols are categorized as topology-based and geographic protocols. In topology-based routing, a route is established between the source and the destination prior to data transmission. This is achieved by maintaining routing tables comprising of link information shared among vehicles through the exchange of control packets. Topology-based routing protocols are further classified into proactive, reactive and hybrid. In contrast, geographic routing protocols neither maintain routing tables nor establish routes prior to data transmission. Instead, data packet is forwarded to the next best hop, which is selected based on the information available in a vehicle’s vicinity. These protocols are further classified into sender-based and receiver-based forwarding. The former approach enables the vehicle that sends the data packet to select the next best forwarding vehicle towards destination, while in the latter approach, the sending vehicle broadcasts the data packet and the receiving vehicle decides whether or not to forward the packet.

#### 2.3.2. Challenges

Efficient and robust routing is still a key challenge in VANETs. This is because the developers of the routing protocols have to consider various technical challenges encountered in VANETs [5,6,42,43,44]. Scalability is one of the characteristics of VANETs and poses a significant hurdle to efficient routing, as the protocol needs to adapt to a varying number of vehicles in the network. To cope with the scalability issue, a routing protocol should make routing decisions solely based on information available in its vicinity. Performing localized operations will eliminate the need for a routing protocol to know the topology of the entire network hence resulting in considerably less overhead. Apart from this, due to the dynamic nature of VANETs, most of the topology-based routing protocols result in degraded network performance [42]. Geographic protocols are more suitable for vehicular networks because of their ability to make routing decisions on the fly. Also, these protocols leverage the constrained movement pattern of vehicles on road by using the location information to further optimize the routing decisions. However, sender-based forwarding requires periodic exchange of information among neighboring vehicles in order to enable the sending vehicle to select the next best vehicle to forward the data packet towards destination. This may result in significant overhead. A better alternative is to use receiver-based forwarding schemes where the receiving vehicle decides whether or not to forward the data packet based on the information available in the header of data packet. The approach offers low overhead since there is no need for periodic information sharing among vehicles [45]. However, minimizing duplicate packet transmissions and reducing transmission delay are the key challenges in receiver-based forwarding schemes.

### 2.4. Receiver-Based Data Forwarding

This section discusses the basic idea of receiver-based scheme while also highlighting some of the existing receiver-based routing protocols in VANETs.

#### 2.4.1. Basic Idea

**Figure 1:**
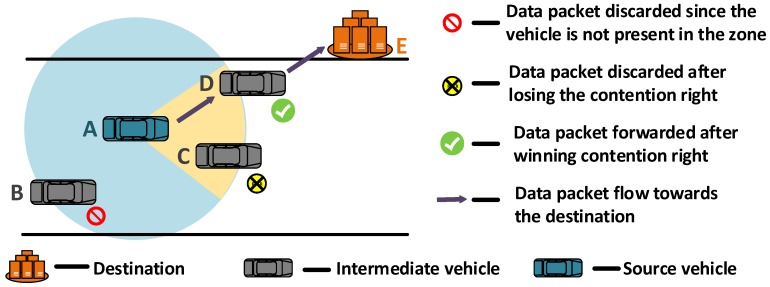
Illustration of a receiver-based forwarding scheme in VANETs.

Figure 1 illustrates the general receiver-based scheme in VANETs. It consists of mainly two features: forwarding zone and waiting time criteria. The forwarding zone, which is highlighted by the yellow region in Figure 1, is the eligibility criterion that every contender checks before forwarding the data packet. If the vehicle is not located within the forwarding zone, as in the case of vehicle B, it discards the data packet. Vehicles located in the forwarding zone contend for the contention right by setting the waiting time based on certain criteria. A vehicle in which the waiting time expires first wins the contention right and broadcasts the data packet further. Other contending vehicles, as in the case of vehicle C, upon hearing the redundant data packet reception discards the data packet.

The purpose of forwarding zone criterion is to minimize the unwanted multiple path formation which occurs when two or more contending vehicles are not located in wireless range of each other [46]. The key to reduce the unnecessary multipath formation is to set the forwarding zone such that all vehicles in the zone are in wireless range of each other.

#### 2.4.2. Related Work

The beacon-less routing (BLR) proposed in [47] is a receiver-based scheme, where the vehicle on reception of data packet uses location information to decide whether or not to participate in the data forwarding process. Three forwarding zones, i.e., circle, sector, and reuleaux triangle, were tested for this protocol. For the waiting time criteria, each contending vehicle assigns a dynamic forwarding delay based on its geographic progress towards destination.

The work in [10] presented another receiver-based routing protocol called video reactive tracking-based unicast (VIRTUS) which is developed to support video streaming in vehicular environment. The forwarding zone is calculated using a forwarding angle θ, and the waiting time criteria is based on the geographic progress towards destination and link stability of the contending vehicle. The link stability is computed based on the current and future location of vehicles. Here, the future locations are predicted using Bayesian state estimation approach. However, VIRTUS requires the source and destination vehicles to periodically exchange information to update their current location coordinates to each other.

Location-aware multipath video streaming (LIAITHON) [45] is a multipath receiver-based routing protocol developed to fulfil video streaming requirements over VANET by distributing the loads over two paths. The two paths are ensured to have the minimum route coupling effect and are selected based on the degree of closeness, geographic progress and link stability. The route coupling between the two paths is avoided by keeping them at least two times of their transmission range apart. Here, multiple paths are used to support high data rate requirements of video streaming. However, multiple paths also lead to redundant data packets flowing throughout the network, which may cause network congestion and packet collision.

The work in [48] further enhanced LIAITHON to LIAITHON+ by increasing the number of multiple paths to three. Although having a greater number of paths may improve load distribution and support higher data rate, but it may also result in increased path length and the route coupling effect. Also, at a higher data rate, too many redundant packets may flow in the network resulting in an increased number of packet collisions.

Besides, the distributed beaconless dissemination (DBD) [11] is a beaconless routing protocol which uses backbone-based approach to maintain robust and high quality routes, thereby enabling live video flows on multimedia highway VANETs for V2V scenarios. DBD does not use forwarding zone criterion as the transmission range of vehicles is greater than the width of the highway roads, which allows the vehicles to hear each other’s data packet transmissions. Also, the vehicles that are traveling in opposite direction with respect to the vehicle broadcasting the data packet do not participate in the contention process. Another feature of DBD is the use of the link-adaptive approach. Specifically, once the contending vehicle wins the contention right, it stays in the “winner” state for a specific duration where it forwards any incoming data packet without setting any waiting time. The duration for which the relaying vehicle stays in the “winner” state is computed based on the location information of the previous and current relaying vehicles, and the wireless range of the vehicles.

## 3. Intersection-Based Link-Adaptive Beaconless Forwarding for City Scenarios

Intersection-based link-adaptive beaconless forwarding for city scenarios (ILBFC) protocol exploits geographic information of road intersections in limiting the duration for which a relay vehicle stays as a default forwarder. Also, an urban road traffic adaptive winner relay management scheme is employed where the relay vehicle when acting as default forwarder decides on every data packet reception whether or not to continue as default forwarder. The details of ILBFC are discussed next.

### 3.1. VANET Model

The vehicles are assumed to be moving in four different directions (east, west, north and south) in an urban environment. The WAVE architecture comprises of IEEE 802.11p and IEEE 1609 standards to support the lower two (PHY and MAC) and higher layers, respectively, is used as the communication model. Here, V2V communication is used to forward the data packet from a source vehicle until the last-hop vehicle, whereas V2I communication is used to forward the packet from the last-hop vehicle to destination (static infrastructure). Each vehicle is assumed to be equipped with GPS and digital maps. The GPS feature enables the vehicle to know its location information, and digital maps further reveal the location information of the destination and the intersections. Apart from this, each vehicle receiving the data packet knows the position information of the previous relay vehicle, which is stored in the header of data packet. Also, for all simulation scenarios, during data transmission, it is assumed that there exists at least one data packet that has reached destination traversing more than one hop. Finally, for the forwarding angle calculations, the line connecting the relaying vehicle and the destination is considered as x-axis. Also, the terms vehicle and node will be used interchangeably and carry the same meaning.

### 3.2. Routing Protocol Design

In ILBFC, the data forwarding process is totally beaconless and all the necessary information required for the relay selection process is stored in the header of data packet. The protocol uses the two-way data-ACK packet exchange instead of the four-way RTS-CTS-data-ACK exchange (RTS, Request To Send; CTS, Clear To Send). Here, only the destination node after receiving the data packet sends the acknowledgement (ACK) packet to the last hop node. Specifically, the header contains the following information: sequence number, source address, destination address, previous relaying vehicle address, location and velocity information of previous relaying vehicle. After transmission of the data packet, the node sets the timer for retransmission timeout ($\gamma_{out}$). If the node does not receive any confirmation before $\gamma_{out}$, it will retransmit the data packet. The confirmation for the source and all the intermediate relay nodes, except the last hop, is the reception of the same data packet. For the last-hop relay node, the confirmation is the reception of an ACK packet from the destination. Also, the maximum number of retransmissions for each data packet is fixed to 3, after which the data packet is discarded. The key features of ILBFC are explained next.

#### 3.2.1. Redundant Packet Elimination

One of the drawbacks of simple data dissemination techniques such as flooding [49] is that the intermediate relay nodes forward duplicate data packets, resulting in redundant data packets flowing throughout the network. This degrades the network performance, especially in dense scenarios due to the high chances of packet collision and network congestion. Flooding also leads to significant overhead and high bandwidth consumption [42]. To minimize this issue in ILBFC, each node upon reception of a new data packet, records the sequence number. This allows a node to distinguish between a new data packet and a duplicate one, thereby discarding the duplicate data packets.

#### 3.2.2. Forwarding Zone

With the intention to further minimize the flow of redundant data packets in ILBFC, a forwarding zone is set such that all nodes located in the zone are in wireless range of each other. To achieve this, a forwarding zone of sector shape with an angle of 60${}^{\circ }$ towards destination is set. A fixed forwarding angle of 60${}^{\circ }$ limits the maximum distance within the forwarding zone to the wireless range of the node. This will enable the nodes in the forwarding zone to hear each other’s packet transmission, thereby allowing only one node in the forwarding zone to relay the data packet. A node is located in the forwarding zone when it satisfies the following condition:

(1) $$2\times arccos\left|\frac{\vec{NS}\cdot \vec{DS}}{\left|\vec{NS}\right|\times \left|\vec{DS}\right|}\right|\leq 60^{\circ },$$

where $\vec{NS}$ is the position vector from the contending node to the previous relay node, and $\vec{DS}$ is the position vector from the previous relay node to the destination. Nodes that do not satisfy this condition do not proceed with the contention process and discard the data packet.

#### 3.2.3. Waiting Time Criteria

Once the node satisfies the forwarding zone criterion, ILBFC determines the suitability of the node to be a relay by setting the waiting time, which is based on the geographic progress towards the destination ($\gamma_{geo}$) and the link stability of the node ($\gamma_{stab}$). $\gamma_{geo}$ for a node is calculated as follows:

(2) $$\gamma_{geo}=1-\frac{\left|\vec{DS}\right|-\left|\vec{DN}\right|}{r},$$

where *r* is the transmission range of the node, and $\vec{DN}$ is the position vector from destination to contending node. $\gamma_{stab}$ is computed using link duration ($\delta_{link}$), which is the minimum duration for which a contending node is in the communication range of the previous relay node. Furthermore, $\gamma_{stab}$ is made to be more adaptive to the urban environment by taking into account the road intersections to limit $\delta_{link}$. Firstly, $\delta_{link}$ is calculated using Equation (3). The sub-variables for Equation (3) are given in the expressions (4)–(8):

(3) $$\delta_{link}=\frac{1}{V}\times \left[-\left(a\times v_{rx}+b\times v_{ry}\right)\pm \sqrt{V\times r^{2}-{\left(a\times v_{ry}-b\times v_{rx}\right)}^{2}}\right],$$

where

(4) $$V={v_{rx}}^{2}+{v_{ry}}^{2},$$

(5) $$v_{rx}=v_{cx}-v_{px},$$

(6) $$v_{ry}=v_{cy}-v_{py},$$

(7) $$a=x_{c}-x_{p},$$

(8) $$b=y_{c}-y_{p},$$

with ($x_{c},y_{c}$) and ($x_{p},y_{p}$) denoting the position coordinates of the contending node and the previous relay node, respectively, and ($v_{cx},v_{cy}$) and ($v_{px},v_{py}$) being the velocity coordinates of the contending node and the previous relay node, respectively. However, nodes with almost the same relative velocities will have a large link duration values, which is unrealistic in city scenarios. This is because city scenarios consist of many road intersections such as traffic junctions and other irregular turns and the direction and speed of the vehicles may change, rendering the previously calculated $\delta_{link}$ inaccurate. Therefore, in ILBFC, the maximum value of $\delta_{link}$ for a vehicle is limited based on the road intersection towards which the vehicle is moving. Specifically, the minimum duration for the vehicle to reach the next road intersection ($\delta_{int}$) is calculated as follows:

(9) $$\delta_{int}=\frac{\sqrt{{\left(x_{i}-x_{c}\right)}^{2}+{\left(y_{i}-y_{c}\right)}^{2}}}{v_{max}},$$

where ($x_{i},y_{i}$) are the position coordinates of the next intersection, and $v_{max}$ is maximum speed of the vehicle. The effective link duration ($\delta_{eff}$) incorporating the road intersection information is calculated as follows:

(10) $$\delta_{eff}=\left\{\begin{array}{cc} \delta_{link}, & \mathrm{if}\;\delta_{link}\leq \delta_{int}\\ \delta_{int}, & \mathrm{otherwise}. \end{array}\right.$$

Once $\delta_{eff}$ is known, $\gamma_{stab}$ can then be computed as follows:

(11) $$\gamma_{stab}=1-\frac{\delta_{eff}}{\Lambda },$$

where Λ is the maximum reservation time and is computed as follows:

(12) $$\Lambda =\frac{d_{max}}{v_{max}}.$$

Here, $d_{max}$ is the maximum distance of the road between two road intersections. Finally, the waiting time (γ) as mentioned in [10] is calculated as follows:

(13) $$\gamma =[\alpha \times \gamma_{geo}+\left(1-\alpha \right)\times \gamma_{stab}]\times \Gamma ,$$

where α is the weighing factor and Γ is the maximum waiting time. Apart from this, nodes also wait for an additional random delay (β) to avoid situations where multiple nodes get a very similar γ.

#### 3.2.4. Winner Relay Management

Another issue with the most of receiver-based scheme is the high ETE delay, which is due to the waiting time required for the relay selection procedure for every data packet. In ILBFC, once the relay node wins the contention right, it changes its state to “winner”. The relay node in this state does not participate in forwarding zone and waiting time criteria, and directly forwards all incoming data packets for a duration of $\delta_{eff}$ s. In addition, the relay node stays in this state as long as its distance to the destination ($d_{c}$) is less than the distance between the previous relay node and the destination ($d_{p}$). However, if $d_{c}$≥$d_{p}$, the relay node leaves its “winner” state and discards the data packet. This condition improves the routing efficiency, especially in the city scenarios where the speed and direction changes in a vehicle are relatively high when compared to highway scenarios.

#### 3.2.5. Loop Protection

In urban environment, especially in the presence of streets with loop formation (circular roads), there is chance of having multiple relay nodes in winner states. The proposed ILBFC uses hop-count information to minimize this issue. A relay node in winner state upon reception of a duplicate packet which it is still processing, checks the hop-count information in the packet header before discarding it. If the hop-count of the packet ($H_{new}$) is much greater than the one (*H*) it is still processing (i.e., $H_{new}\geq H+\eta$), it implies that there is another relay node that has already forwarded the very same packet. Here, η is a constant greater than zero. Hence, the current relay node leaves its “winner” state and discards the packet. Apart from this, ILBFC sets a maximum hop limit ($H_{max}$) that a data packet can traverse and after which it is discarded by the node.

**Figure 2:**
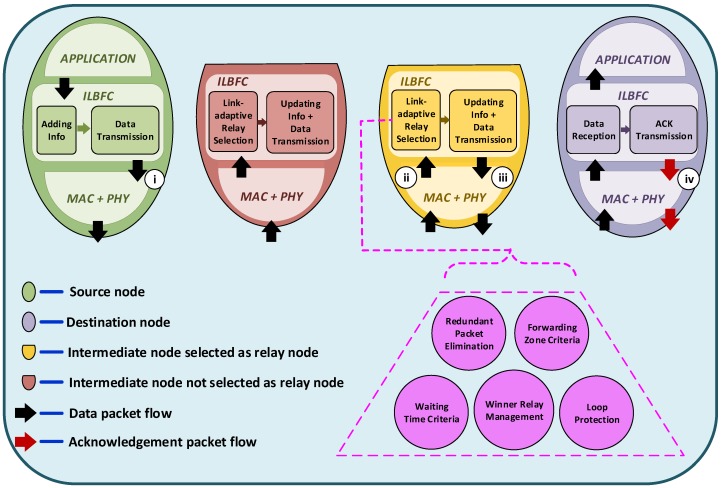
ILBFC architecture.

Figure 2 illustrates the ILBFC architecture and some of its important steps which are as follows: (i) when the source node has a data packet to transmit, ILBFC adds relevant information such as node’s location and mobility information, sequence number, etc., to the header of data packet before broadcasting the packet; (ii) upon reception of the data packet, ILBFC determines the suitability of the relay node by checking the above mentioned conditions in order to schedule the packet for further forwarding; (iii) after waiting timer expiration or during default forwarding in case the relay node is in “winner” state, it updates the header of data packet adding relevant information and then broadcasts the data packet; (iv) upon data packet reception at the destination node, an ACK packet is transmitted to enable the last hop relay node to cancel its timer.

## 4. Performance Analysis

In this section, the performance of ILBFC is compared with other existing state-of-the-art routing protocols. The details of scenario setup, performance metrics used, and simulations tests are discussed next.

### 4.1. Scenario Setup and Performance Metrics

**Figure 3:**
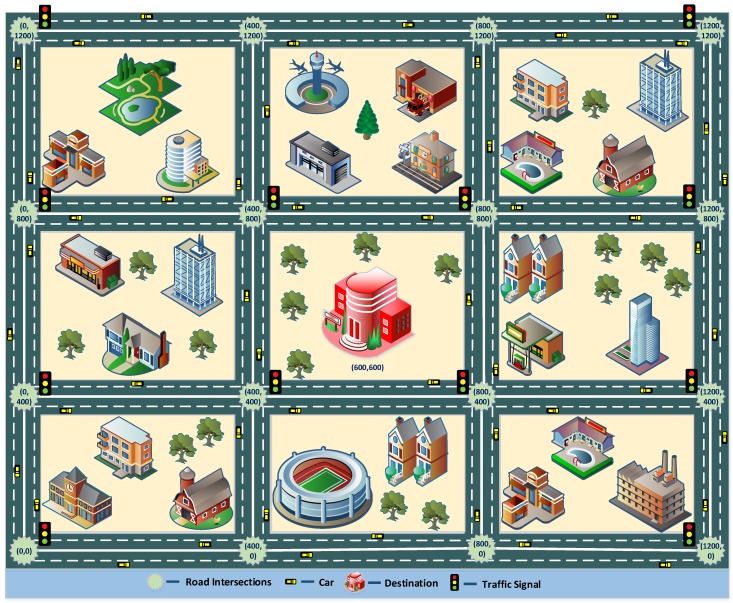
Illustration of the scenario.

With an aim to replicate urban environment, a city scenario in the form of Manhattan grid is created using a software called simulation of urban mobility (SUMO) [50]. Figure 3 shows an illustration of a scenario highlighting the position coordinates of road intersections and destination.

As depicted in the figure, the roads are bidirectional with each direction consisting of two lanes. In addition, to make the scenario more realistic, traffic signals were installed at some of the road intersections. Mobility traces generated from SUMO are then imported in QualNet 8.1 network simulator [51] through mobility model generator for vehicular networks (MOVE) [52] tool for performance analysis.

Also, in order to simulate a realistic vehicular wireless channel, the path loss must be beyond basic path loss estimation models such as free-space path loss [53] as the radio propagation needs to take into account the different sources of interference. One such path loss model is the two-ray interference model [54] which considers the constructive/destructive self-interference while taking into account the transmitter ($h_{t}$) and receiver ($h_{r}$) antenna heights over the ground and the relative permittivity ($\varepsilon_{r}$) of the ground surface. The path loss ($L_{t}$) for two-ray interference model mentioned in [54,55] is calculated using Equation (14). The sub-variables for Equation (14) are given in the expressions (15)–(21):

(14) $$L_{t}=20\times \log(4\times \pi \times \frac{d}{\lambda }\times |1+\left(\Upsilon_{⊥}\times e^{j\times \varphi }\right){|}^{-1}),$$

(15) $$\varphi =2\times \pi \times \frac{d_{ref}-d_{los}}{\lambda },$$

(16) $$\Upsilon_{⊥}=\frac{\sin\theta_{i}-\sqrt{\epsilon_{r}-\cos^{2}\theta_{i}}}{\sin\theta_{i}+\sqrt{\epsilon_{r}-\cos^{2}\theta_{i}}},$$

(17) $$\epsilon_{r}=\varepsilon_{r}-\left(j\times 60\times \mu \times \lambda \right),$$

(18) $$d_{los}=\sqrt{d^{2}+{\left(h_{t}-h_{r}\right)}^{2}},$$

(19) $$d_{ref}=\sqrt{d^{2}+{\left(h_{t}+h_{r}\right)}^{2}},$$

(20) $$\sin\theta_{i}=\frac{h_{t}+h_{r}}{d_{ref}},$$

(21) $$\cos\theta_{i}=\frac{d}{d_{ref}},$$

where *d* is the distance between the transmitting and receiving antennas, λ is the wavelength, $\Upsilon_{⊥}$ is the reflection coefficient, and φ is the phase difference. $d_{los}$ and $d_{ref}$ are the distances covered by direct and reflected rays, respectively. *j* represents the complex number ($\sqrt{-1}$) and $\theta_{i}$ is the angle of incidence for reflected ray, where subscript *i* indicates “incidence". As mentioned in [55], the material generally used for road surface is asphalt and has a relative permittivity value ($\varepsilon_{r}$) of 5 and a conductivity value (μ) of 0. Although two-ray interference model offers increased level of realism compared to the free-space model for unobstructed scenarios, it does not consider the shadowing effect due to obstructions such as buildings, trees, etc. Therefore, in the proposed scenario, the total path loss (*L*) is computed as follows:

(22) $$L=L_{t}+L_{sys}+X_{\sigma },$$

where $X_{\sigma }$ is a zero mean Gaussian distributed random variable with σ being the standard deviation. The Gaussian distribution is used to take into account the random effects of shadowing that a moving vehicle may face from the surrounding obstructions. $L_{sys}$ represents the system loss that occurs during the data transmission at the sender side and the data reception at the receiver side, and is computed as follows:

(23) $$L_{sys}=A_{eff}+A_{mismatch}+A_{connection},$$

where $A_{eff}$ is the loss due to antenna efficiency, $A_{mismatch}$ is the antenna mismatch loss, and $A_{connection}$ is the antenna connection loss. Here, all the variables of Equations (22) and (23) are given in dB.

The key simulation parameters used for evaluating the results are highlighted in Table 2. Although the duration of entire simulation is 300 s, there is no data packet exchange between the nodes for the first 200 s and the only event during this time is node movement, followed by 100 s of data packet exchange between the nodes. This is done to avoid cold start issues in the results.

**Figure 4:**
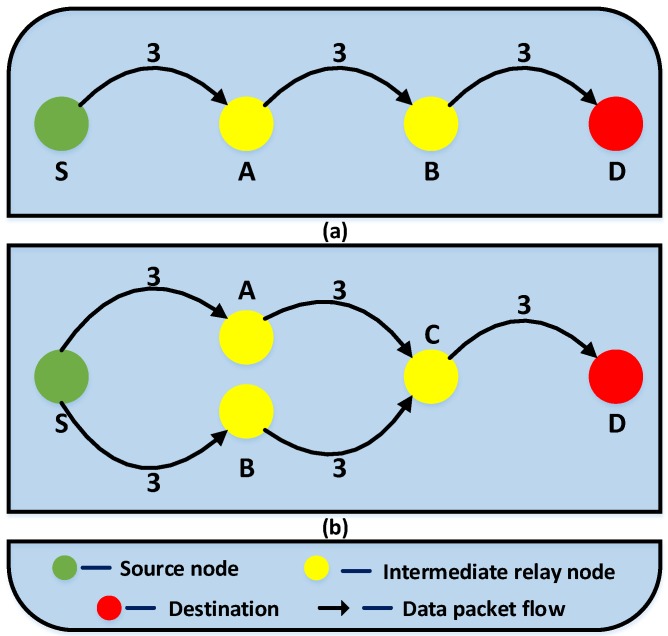
Illustration of data packet transmission scenario: (**a**) perfect unicast transmission and (**b**) data packet transmission with multiple relay nodes at single hop level.

For evaluating the network performance, three performance metrics are considered. The PDR is the ratio between the number of packets received at the destination and the total number of packets sent by the source node. The average ETE delay refers to the average time taken for a data packet to be transmitted across a network from source node to destination. In addition to PDR and average ETE delay, the PRC is proposed to give a measure of unicast capability of a receiver-based routing scheme. Most of the receiver-based schemes try to minimize multiple relay nodes at single hop level by discarding duplicate packets, by using forwarding zone and assigning waiting time. However, in some situations such as when the waiting time of multiple nodes is similar, there are chances of having multiple relay nodes, thereby leading to multiple path formation in the network. By accounting for PRC, the ability of a routing protocol to suppress unnecessary multiple relay nodes at single hop level can be determined. PRC is calculated as follows:

(24) $$\mathrm{PRC}=\frac{\sum_{i=1}^{n}N_{ifd}}{\left(H_{ave}-1\right)\times N_{rec}}-1,$$

where $N_{ifd}$ is the the total number of data packets forwarded by intermediate relay node *i*, and $\sum_{i=1}^{n}N_{ifd}$ refers to the summation of total number of data packets forwarded by all *n* intermediate relay nodes. $H_{ave}$ is the average number of hops that a data packet traverses before reaching destination and $N_{rec}$ is the total number of data packets received at destination. Here, single hop communication involving only source and destination is excluded while calculating $H_{ave}$ and $N_{rec}$. A PRC value of 0 implies a perfect unicast operation while any value greater than 0 represents the presence of multiple relay nodes at single hop level. Figure 4 illustrates two different scenarios of data packet transmission for a better understanding of the PRC metric.

Figure 4a shows the perfect unicast transmission case. Here, each node is in the wireless range of two nodes at maximum (one node forward and one node backward). As depicted in the figure, the source node broadcasts 3 data packets which are then forwarded by the intermediate relay nodes (A and B), resulting in a total of 6 data packets being relayed by the intermediate nodes. These data packets are then received at the destination after traversing an average of 3 hops. Upon computing PRC for this scenario using Equation (24), a PRC value of 0 is obtained. On the other hand, for the data packet transmission scenario shown in Figure 4b, two intermediate nodes (A and B) at the same hop level relay the data packet, thereby resulting in 3 redundant data packets being forwarded to node C. Although assuming that node C discards the redundant data packets, it still leads to a total of 9 data packets being forwarded by the intermediate relay nodes, resulting in a PRC value of 0.5. A greater PRC value implies formation of more multiple relay nodes at single hop level referring to the inefficient unicast operation of the routing protocol.

### 4.2. Protocol Specification

Table 3 highlights various protocol parameters of ILBFC. The performance of ILBFC is compared with other existing state-of-the-art routing protocols. The specification of these protocols is described next.

#### 4.2.1. Link-Adaptive Beaconless Forwarding

Link-adaptive beaconless forwarding (LBF) protocol is a modified version of ILBFC, and it does not contain road adaptive features present in ILBFC. Here, $\delta_{eff}=\delta_{link}$ and is computed using Equation (3) with maximum reservation time (Λ) value set to 100 s which represents the maximum duration for the data packet exchange among nodes in the simulated scenario. Also, the relay node stays in the “winner” state and forwards all the incoming data packet for an entire duration of $\delta_{link}$ s since there is no winner relay management condition ($d_{c}$≥$d_{p}$) as present in ILBFC. The rest of the features (redundant packet elimination, forwarding zone and loop protection) of LBF are similar to ILBFC. The performance of LBF is compared with ILBFC to analyze the impact of location information of road intersections and urban road traffic adaptive winner relay management on the overall routing and network performance.

#### 4.2.2. Distributed Beaconless Dissemination

Distributed beaconless dissemination (DBD) protocol performs receiver-based dissemination and is specifically built to transfer video data in highway scenarios [11]. The scheme does not have a forwarding zone criterion, since the transmission range of vehicles is usually greater than the width of the highway roads and, hence, they are able to notify their data packet transmission to other vehicles. However, the vehicles that are traveling in opposite direction with respect to the sender discard the received data packet and do not take part in the contention. For the waiting time criterion, a node after receiving data packet sets the waiting time solely based on its geographic progress towards the destination, which is as follows:

(25) $$\gamma =\gamma_{geo}\times \Gamma ,$$

where $\gamma_{geo}$ is calculated according to Equation (2) and Γ is set to 50 ms which is the same value as used in ILBFC and is indicated in Table 3. For the winner relay management, the relay node stays in the “winner” state until its distance from the sender (*d*) is such that 0.75 ×r≤d≤r. The rest of the features (redundant packet elimination and loop protection) are similar to ILBFC. Performance of DBD is analyzed to test the compatibility of a receiver-based routing scheme developed for highways in an urban scenario.

#### 4.2.3. Beacon-Less Routing

The beacon-less routing (BLR) protocol [47] is a simple receiver-based scheme where the nodes on reception of data packet, based on their presence in forwarding zone decide whether or not to contend for the forwarding right of the data packet. Here, the forwarding zone set is the same as that of ILBFC. Furthermore, all the eligible nodes then assign a waiting time solely based on their geographic progress towards destination. Apart from this, an additional β similar to ILBFC is set to avoid redundant data packet forwarding by multiple relay nodes with similar γ. However, BLR does not contain any winner relay management feature, and the intermediate nodes perform relay selection process (forwarding zone and waiting time criteria) for every data packet received. For the loop protection, only the maximum hop limit ($H_{max}$) feature is considered in BLR. Since BLR performs relay selection for every received data packet, it will be interesting to compare its performance with ILBFC, LBF and DBD. The latter routing techniques after the contention phase, allows the selected relay node to forward all the incoming data packets for a specific time based on their link duration value.

### 4.3. Simulation Results

The simulations are carried out by varying source transmission rate, node density, and maximum node velocity. The simulation results for these three cases are discussed next.

#### 4.3.1. Source Transmission Rate Variation

**Figure 5:**
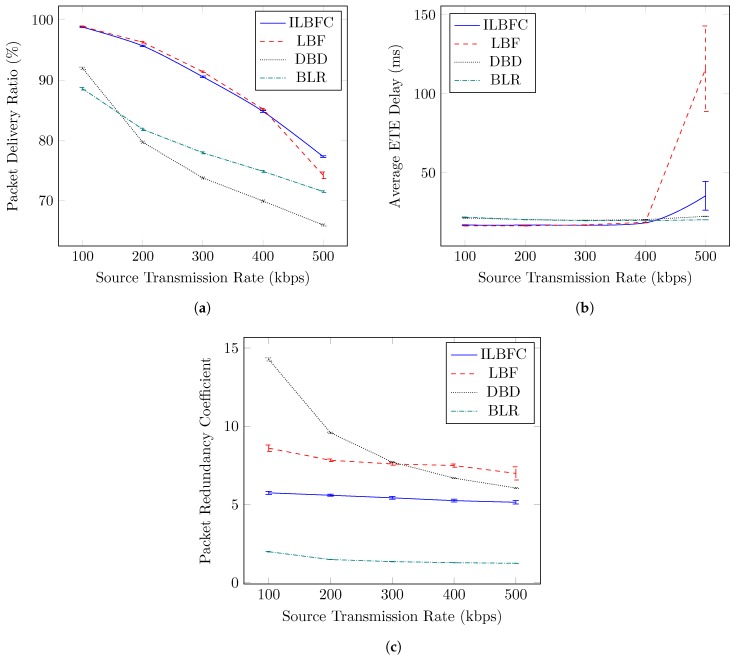
Performance comparison of ILBFC with LBF, DBD and BLR versus source transmission rate while keeping the maximum speed of the node equals 50 km/hr and the number of nodes equals 40. (**a**) PDR versus source transmission rate; (**b**) Average ETE delay versus source transmission rate; (**c**) PRC versus source transmission rate.

This section shows the impact of source transmission rate on the performance of the protocols in terms of PDR, average ETE delay and PRC. The transmission rate of a source node ($R_{src}$) is defined as follows:

(26) $$R_{src}=S_{bits}/T_{intarr},$$

where $S_{bits}$ is the data packet size in bits and $T_{intarr}$ is the inter-arrival time of the data packets. Figure 5 highlights the impact of source transmission rate variation on the performance of different routing protocols.

Specifically, $R_{src}$ is varied from 100 kbps to 500 kbps with an incremental step of 100 kbps. Also, for this scenario, the maximum speed of the node is set to 50 km/hr, and the number of nodes (one static destination and the rest are mobile nodes) is fixed to 40.

Figure 5a shows the PDR performance of the protocols with respect to varying $R_{src}$. BLR protocol at $R_{src}$ equals 100 kbps yields the lowest PDR of all. This is because every received data packet that satisfies the forwarding zone condition has to wait a certain duration depending upon the waiting time criteria before it can further forward the packet. This results in a large number of data packets being queued in buffer and may further lead to data packet being dropped once the buffer is full. Therefore, with increasing $R_{src}$ more data packets are dropped, thus resulting in decreased PDR. The DBD protocol, on the other hand, although it performs better than BLR protocol at $R_{src}$ equals 100 kbps, its performance drops drastically with increasing $R_{src}$. The major reason for this is due to its stringent winner relay management conditions. Unlike highways, where the relative velocity among the vehicles is quite stable, in an urban environment there exist high relative velocity fluctuations among vehicles. Hence, it will be difficult for relay nodes to satisfy the winner relay management condition (0.75 ×r≤d≤r). Therefore, for most of the received data packets, the operation of DBD will be similar to BLR but without forwarding zone, which will further increase packet collision, thereby degrading the PDR performance. Also, ignoring the nodes moving in opposite direction for contention process may reduce the number of suitable contenders for forwarding the data packets. ILBFC and LBF show relatively high PDR performance due to their winner relay management condition which is based on the link duration among vehicles, thereby more data packets are forwarded in “winner” state resulting in fewer data packets being dropped due to buffer overflow. However, LBF at high source transmission rate ($R_{src}$ equals 500 kbps) performs worse than ILBFC because of its lack of road adaptive features. Inaccurate $\delta_{link}$ can lead to relay node staying in “winner” state for too long. This increases the chances of relay node moving out of the forwarding zone, thereby resulting in multiple relay nodes forwarding redundant data packet throughout the network, hence leading to higher chances of packet collision. Also, another drawback of relay node in the “winner” state moving out of the forwarding zone is the fact that it may travel in a direction where there are either none or very few suitable contending nodes available to further forward the data packet towards destination. In contrast, ILBFC limits $\delta_{link}$ based on the next nearest intersection to more accurate $\delta_{eff}$ while also checking the relative position of relay node in the “winner” state with respect to previous sender. This ensures that “winner” relay node does not move in the wrong direction, while also reducing the chances of the “winner” relay node moving out of the forwarding zone, hence resulting in superior PDR performance when compared to rest. For this scenario, the average PDR performance gains of ILBFC over LBF, DBD and BLR are 0.2%, 13.2% and 10.5%, respectively.

Figure 5b depicts the average ETE delay of the data packets received at the destination. For most of the source transmission rate variation cases (100 kbps $\leq R_{src}\leq$ 400 kbps), both ILBFC and LBF yield better performance when compared to DBD and BLR. This is due to their better winner relay management based on the link duration, thereby minimizing the delay caused by the relay selection process. Specifically, for the source transmission rate variation cases (100 kbps $\leq R_{src}\leq$ 400 kbps), the mean values of the average ETE delay of ILBFC, LBF, DBD, and BLR are 17.3 ms, 17.1 ms, 20.5 ms and 20.4 ms, respectively. At higher source transmission rate ($R_{src}=500$ kbps), the low average ETE delay of DBD and BLR is simply because of their degraded PDR performance highlighted in Figure 5a, as an increase in $R_{src}$ results in more data packets being dropped due to buffer overflow. As far as LBF and ILBFC are concerned, LBF at $R_{src}$ = 500 kbps results in relatively high average ETE delay when compared to ILBFC, which is due to multiple relay nodes forwarding the same data packet at single hop level leading to redundant data packets flowing throughout the network and causing packet collision and network congestion. This happens because of the relay nodes moving out of the forwarding zone while still being in the “winner” state. Also, these relay nodes may further travel in a direction where there are either none or very few suitable relay node contenders to further forward the packet, thereby resulting in longer routes to destination causing high average ETE delay. ILBFC, on the other hand, performs better than LBF while also offering better PDR performance due to its accurate $\delta_{eff}$ estimation which is based on the location information of road intersections. This results in less longer routes to destination. Also, at single hop level, the chances of more than one relay node being selected to forward the data packet is reduced, hence yielding lower average ETE delay.

Figure 5c shows the PRC performance of protocols accentuating their efficiency in terms of forwarding the data packet in unicast way with respect to varying source transmission rate. With an average PRC value of 8.9, DBD results in the highest PRC indicating low unicast efficiency. The major reason for this is its lack of forwarding zone criteria, which leads to multiple relay nodes being formed at single hop level. The decrease in PRC for DBD with increasing $R_{src}$ is simply because of the increase in the number of packets being dropped by the intermediate relay nodes due to packet collision. In contrast, with an average PRC value of 1.5, BLR yields the lowest PRC, implying high unicast efficiency. One of the main reasons is that unlike others, BLR performs relay selection for every data packet received, thereby minimizing the multiple relay nodes at single hop level. Another reason is the high packet drops encountered at intermediate relay nodes due to buffer overflow. This further results in less data packets being forwarded and hence contributes towards low PRC. The impact of using the road intersection information in correctly estimating link duration on PRC performance can be clearly seen when comparing LBF and ILBFC. Due to its accurate $\delta_{eff}$ estimation, the ILBFC protocol which has an average PRC value of 5.4, ensures relatively less multiple relay node formation at single hop level when compared to LBF which has an average PRC value of 7.7, thereby resulting in lower PRC.

#### 4.3.2. Node Density Variation

**Figure 6:**
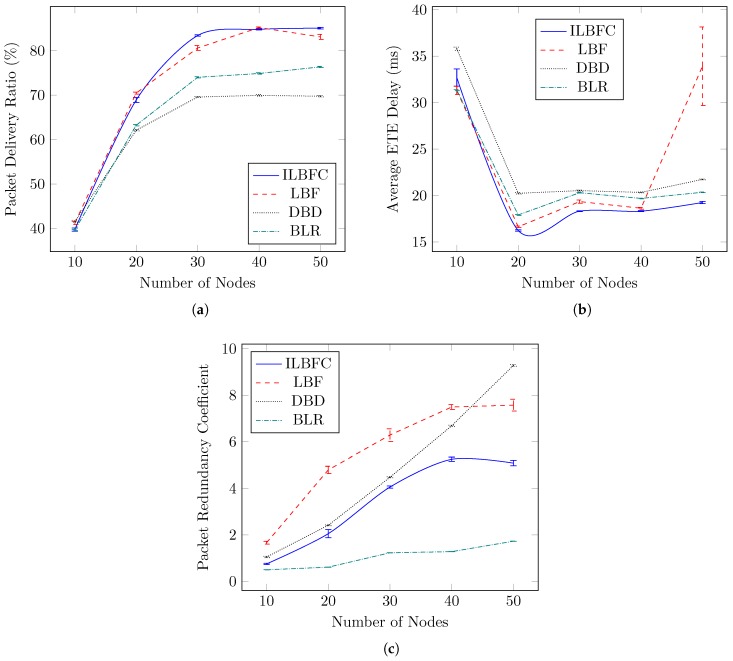
Performance comparison of ILBFC with LBF, DBD and BLR versus number of nodes, while keeping the maximum speed of the node equals 50 km/hr and the source transmission rate equals 400 kbps. (**a**) PDR versus number of nodes; (**b**) Average ETE delay versus number of nodes; (**c**) PRC versus number of nodes.

This section demonstrates the influence of node density variation on the performance of the routing protocols in terms of PDR, average ETE delay and PRC as shown in Figure 6. Node density is considered high when a large number of nodes are in proximity of one another within a particular area and vice versa for low node density scenarios. Here, five scenarios obtained by varying the number of nodes from 10 to 50 with an incremental step of 10 have been tested with a fixed simulation area of 1200 m × 1200 m as indicated in Table 2. In all network density scenarios, except one static destination, all nodes are mobile. Also, for these scenarios, the maximum speed of the node is set to 50 km/hr and $R_{src}$ is fixed to 400 kbps.

Figure 6a depicts the PDR performance of the protocols with respect to varying node density. It can be seen that PDR for all the protocols is increasing until the number of node reaches 30, after which it remains almost stable. The major reason for the packet drops in low density scenarios is the lack of availability of suitable relay nodes to forward the data packet towards destination. DBD has the lowest PDR performance due to its lack of forwarding zone criteria which may lead to packet collision and its strict winner relay management condition which rarely allows the relay node to be in “winner” state. This further results in packet drops due to buffer overflow, which is one of the drawbacks of frequent relay selection process and is also witnessed in the BLR’s PDR performance. ILBFC yields superior PDR performance compared to other protocols due to its better winner relay management based on $\delta_{eff}$ which enables the relay nodes to stay in “winner” state but not for too long as in the case of LBF, possibly reducing packet collision. The average PDR performance gains of ILBFC over LBF, DBD and BLR for the case when the number of nodes is varied from 30 nodes to 50 nodes are 1.5%, 14.7% and 9.4%, respectively.

The average ETE delay performance of the data packets received at the destination for all the protocols with respect to varying node density is shown in Figure 6b. All protocols result in high average ETE delay when the node density is the lowest (10 nodes). The reason for this is the scarcity of suitable relay nodes leading to data packets being sent to destination through longer routes causing high average ETE delay. ILBFC, due to its better winner relay management, has the lowest average ETE delay when compared to the rest. Specifically, for this scenario, the mean values of the average ETE delay of ILBFC, LBF, DBD, and BLR are 21 ms, 24 ms, 23.7 ms and 21.9 ms, respectively. Another noticeable aspect of the analysis is the LBF’s large average ETE delay at high node density (50 nodes), which is because of the network congestion caused by the redundant packets flowing throughout the network. This in turn is due to selection of more that one relay node at the single hop level which is one of the drawbacks of “winner” relay node moving out of the forwarding zone.

Figure 6c shows the effect of node density variation on the PRC performance. It can be seen that there is an overall increase in PRC, which corresponds to decreasing unicast efficiency, for all the protocols with rising node density. This is expected since the upsurge in the number of nodes increases the chances of having multiple relay nodes at single hop level. BLR with an average PRC value of 1.1, minimizes the selection of multiple relay nodes at the single hop level and is due to its relay selection process for every received data packet. Here, DBD with an average PRC value of 4, does not have the highest PRC compared with the rest of all node densities. This is because of the high packet collision and packet drops due to buffer overflow at intermediate relay nodes resulting in fewer data packets being forwarded by intermediate relay nodes. This further reduces the chances of formation of multiple relay nodes at single hop level leading to low PRC. Again, the significance of road intersection information in enhancing the unicast efficiency can be seen when comparing LBF and ILBFC performance. ILBFC which has an average PRC value of 2.9, with its better winner relay management based on $\delta_{eff}$, results in relatively lower PRC with rising node density as compared to LBF which has an average PRC value of 4.6.

#### 4.3.3. Maximum Node Velocity Variation

**Figure 7:**
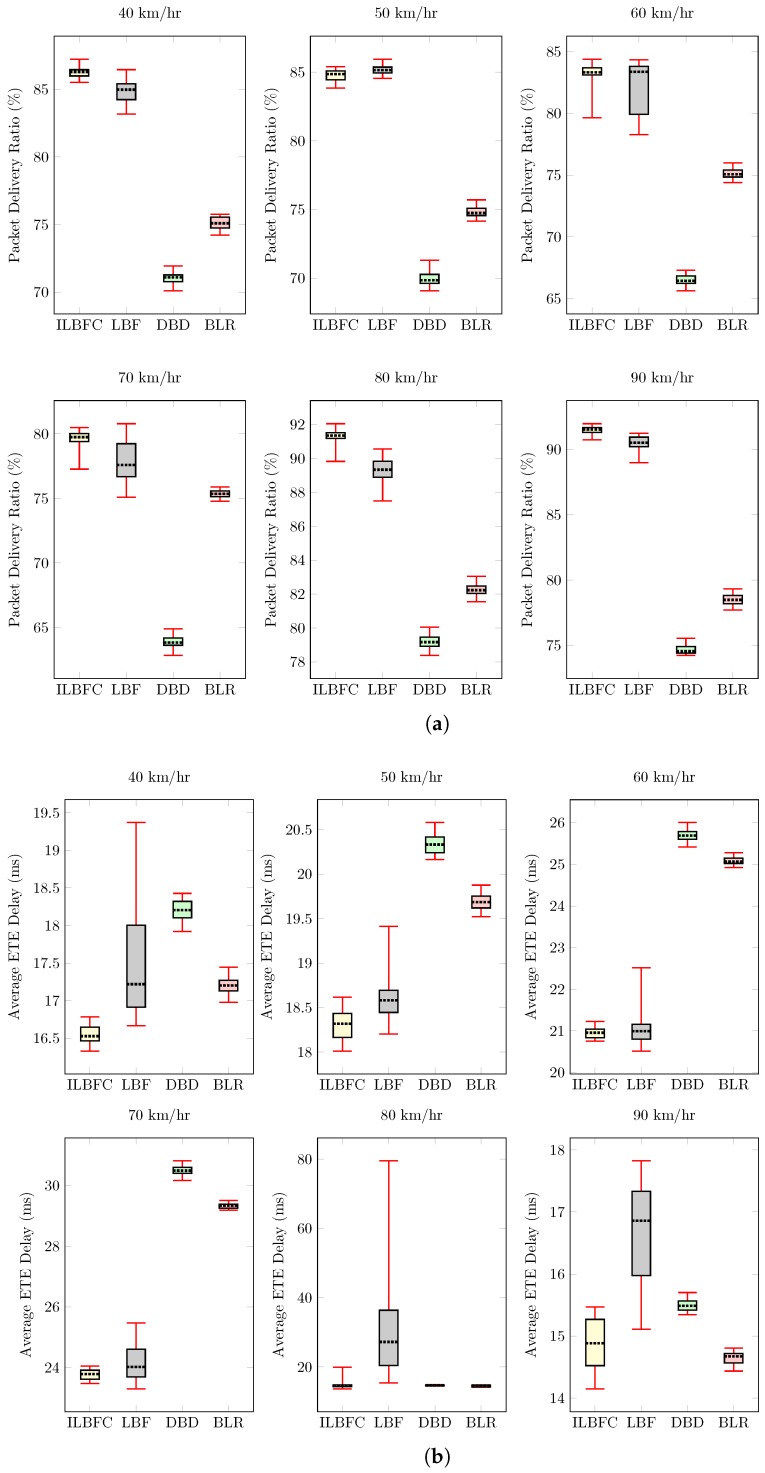

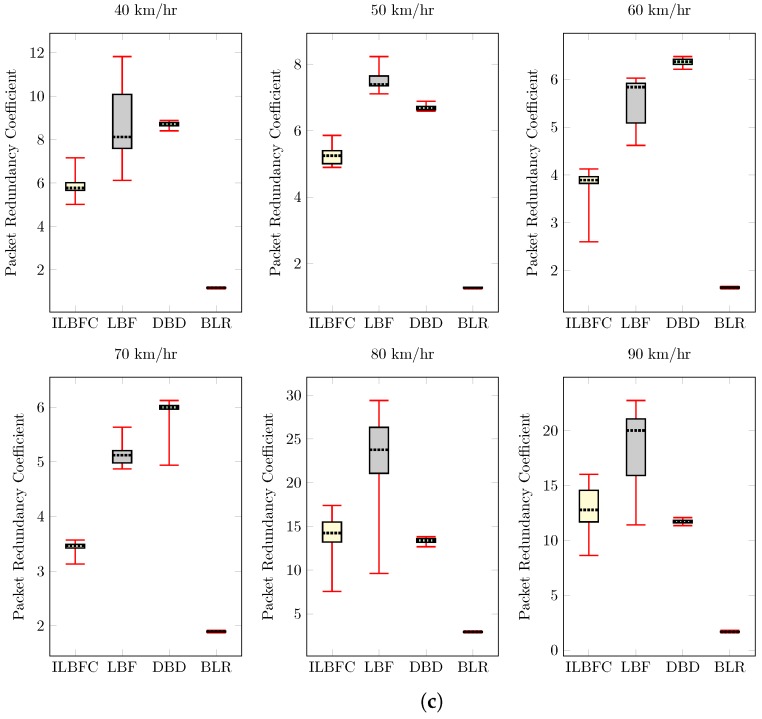
Performance comparison of ILBFC with LBF, DBD and BLR versus maximum node velocity while keeping the source transmission rate equals 400 kbps and the number of nodes equals 40 (cont.). (**a**) PDR versus maximum node velocity; (**b**) Average ETE delay versus maximum node velocity; (**c**) PRC versus maximum node velocity.

This section discusses the performance of routing protocols at different maximum node velocities. Unlike highway and rural scenarios, the maximum speed of the node does not have a direct impact on the performance of routing protocols in an urban environment. This is because of the traffic signals located at various intersections due to which there is a chance that a node with high maximum node velocity may end up having less overall movement during the entire simulation when compared to node having relatively lower maximum node velocity. Hence, to analyze the network performance, instead of using line plots (used in Section 4.3.1 and Section 4.3.2), individual box plots highlighting the performance of all routing protocols are plotted for different maximum node velocity conditions as shown in Figure 7.

Specifically, six maximum node velocity conditions varying from 40 km/hr to 90 km/hr with an incremental step of 10 km/hr are tested. Also in all the conditions, the source node starts moving from the same location. Apart from this, $R_{src}$ and node density for all the conditions are fixed to 400 kbps and 40, respectively.

Figure 7a shows the PDR performance of all routing protocols at different maximum node velocity conditions. ILBFC due to its accurate $\delta_{eff}$ and better relay management conditions gives the highest PDR performance followed by LBF. DBD due to its lack of forwarding zone and strict winner relay management conditions results in both packet drops and packet collision leading to low PDR. For this scenario, the average PDR performance gains of ILBFC over LBF, DBD and BLR are 1.2%, 15.2% and 9.2%, respectively. Figure 7b depicts the average ETE delay of the received data packets at destination for different maximum node velocity conditions. It can be seen that ILBFC offers superior performance having the lowest ETE delay. The mean values of average ETE delay at all maximum node velocities for ILBFC, LBF, DBD and BLR are 18.2 ms, 21.8 ms, 20.8 ms and 20 ms, respectively. The larger ETE delays for LBF in some cases is due to its $\delta_{link}$ which lets its relay node stay in the “winner” state for too long. This results in packets being forwarded in wrong direction leading to an increase in average ETE delay of received data packets. DBD and BLR performs worse than ILBFC while also offering poor PDR performance. Figure 7c highlights the unicasting efficiency of all routing protocols in terms of PRC for different maximum velocity conditions. BLR with an average PRC value of 1.8, due to its relay selection process for every data packet received, ensures the lowest PRC implying high unicast efficiency. However, this comes at the expense of poor PDR and average ETE delay performance. ILBFC with an average PRC value of 7.5, results in relatively lower PRC when compared to DBD (average PRC value of 8.8) and LBF (average PRC value of 11.4) while also having superior PDR and average ETE delay performance than the rest. The impact of incorporating knowledge of road intersections during $\delta_{eff}$ computation and better winner relay management on unicasting efficiency can be clearly observed when comparing the PRC performance of ILBFC and LBF. LBF due to its lack of road intersection knowledge and ineffective winner relay management results in higher PRC inferring lower unicast efficiency.

## 5. Conclusions

Receiver-based data forwarding protocols are well suited to enable remote monitoring applications in urban VANETs. However, they often result in high ETE delay due to frequent relay selection process performed for every data packet. Although the existing work has focused on alleviating this issue by allowing the relay vehicle to stay as a default forwarder for certain duration, the latter duration is not accurate enough since it does not consider various aspects of urban traffic such as the presence of road intersections and drastic speed decay in vehicles. This article proposes a receiver-based data forwarding protocol called ILBFC that makes use of geographic information of road intersections to effectively decide the duration for which a relay vehicle will stay as a default forwarder. Additionally, a winner relay management scheme is used to check the validity of relay vehicle acting as a default forwarder on every data packet reception. This is based on the relative geographic progress of current and previous relay vehicles towards destination. The performance of ILBFC has been compared with LBF, DBD and BLR, in terms of PDR, average ETE delay and PRC for three different scenarios.

The simulation results highlight the performance improvement of ILBFC over LBF, DBD and BLR in terms of PDR, average ETE delay and PRC. This is due to the effective link duration computation of ILBFC which is based on the position coordinates of the upcoming road intersection. Also, ILBFC is equipped with a better winner relay management scheme that reduces the frequency of relay selection process while also taking into account the drastic speed decay in vehicles. In case of PRC performance, BLR offers highest unicast efficiency (implying low PRC) as it performs relay selection process on every data packet reception. However, this comes at the expense of poor PDR performance, which is one of the drawbacks of frequent relay selection and is caused due to buffer overflow. Specifically, the range of PDR performance losses in BLR when compared to ILBFC is between 9–11% for all the three cases. On the other hand, ILBFC offers relatively lower PRC (implying higher unicast efficiency) when compare to LBF and DBD while also giving superior PDR and average ETE delay performance than the rest. Overall, ILBFC provides an efficient and reliable routing solution to enable remote monitoring applications over urban VANETs.

In future work, further simulations under several complex urban scenarios will be carried out to analyze the performance of ILBFC. Also, ILBFC will be extended to provide routing solutions to applications involving mobile destination in urban VANETs. This would require periodic updates from destination vehicle informing other vehicles about its current location. In addition, this may also need the source and other relay vehicles to make use of tracking mechanism to estimate the latest location of the destination vehicle. Apart from this, various physical characteristics of the wireless environment such as signal strength and interference will be incorporated in the relay selection process of ILBFC and their impact will be thoroughly analyzed.

## Figures and Tables

**Table 1:** Data rate requirements to enable remote monitoring applications for different sensors.

Sensor	Signal	Data Rate
Physiological [35,36]	ECG	24–288 kbps
	EEG	43.2 kbps
	EMG	320 kbps
	EOG	1.2 kbps
	EDA	1.2 kbps
	Heart Rate	2–5 kbps
	Blood Pressure	2–5 kbps
	Respiration	800 bps
Audio [35]	Voice	4–25 kbps
	Sound Diagnostic	32–256 kbps
Camera	Images [37]	200 kbps
	Video Telephony [38]	32 kbps
	Video Streaming [39,40,41]	100–500 kbps

**Table 2:** Simulation parameters.

Parameter	Value
Area	1200 m × 1200 m
Simulation time	300 s
Number of simulation runs	30
Transmission range	450 m
Transmission power	29.3 dBm
Channel frequency	5.9 GHz
MAC, PHY parameters	IEEE 802.11p
Data traffic	CBR
Data packet size	512 B
Buffer size	51.2 kB
Nominal bandwidth	27 Mbps
Antenna type	Omnidirectional
Antenna height	1.895 m
$A_{eff}$	0.9691 dB
$A_{mismatch}$	0.3 dB
$A_{connection}$	0.2 dB
Pathloss model	Two ray interference
Shadowing effect, σ	4 dB
Receiver sensitivity	−71.65 dBm

**Table 3:** ILBFC protocol parameters.

Parameter	Value	Parameter	Value
Γ	50 ms	η	3
$\gamma_{out}$	50 ms	$H_{max}$	6
α	0.5	β	10 ms
